# Enhancing disease risk gene discovery by integrating transcription factor-linked *trans*-variants into transcriptome-wide association analyses

**DOI:** 10.1093/nar/gkae1035

**Published:** 2024-11-13

**Authors:** Jingni He, Deshan Perera, Wanqing Wen, Jie Ping, Qing Li, Linshuoshuo Lyu, Zhishan Chen, Xiang Shu, Jirong Long, Qiuyin Cai, Xiao-Ou Shu, Zhijun Yin, Wei Zheng, Quan Long, Xingyi Guo

**Affiliations:** Department of Biochemistry & Molecular Biology, University of Calgary, HMRB 231, 3330 Hospital Drive NW, Calgary, AB T2N 4N1, Canada; Department of Neuroscience, School of Translational Medicine, Faculty of Medicine, Nursing and Health Sciences, Monash University, The Alfred Centre, Level 6, 99 Commercial Road, Melbourne, VIC 3004, Australia; Department of Biochemistry & Molecular Biology, University of Calgary, HMRB 231, 3330 Hospital Drive NW, Calgary, AB T2N 4N1, Canada; Division of Epidemiology, Department of Medicine, Vanderbilt Epidemiology Center, Vanderbilt-Ingram Cancer Center, Vanderbilt University School of Medicine, 2525 West End Ave, Nashville, TN 37203, USA; Division of Epidemiology, Department of Medicine, Vanderbilt Epidemiology Center, Vanderbilt-Ingram Cancer Center, Vanderbilt University School of Medicine, 2525 West End Ave, Nashville, TN 37203, USA; Division of Epidemiology, Department of Medicine, Vanderbilt Epidemiology Center, Vanderbilt-Ingram Cancer Center, Vanderbilt University School of Medicine, 2525 West End Ave, Nashville, TN 37203, USA; Division of Epidemiology, Department of Medicine, Vanderbilt Epidemiology Center, Vanderbilt-Ingram Cancer Center, Vanderbilt University School of Medicine, 2525 West End Ave, Nashville, TN 37203, USA; Division of Epidemiology, Department of Medicine, Vanderbilt Epidemiology Center, Vanderbilt-Ingram Cancer Center, Vanderbilt University School of Medicine, 2525 West End Ave, Nashville, TN 37203, USA; Department of Epidemiology and Biostatistics, Memorial Sloan Kettering Cancer Center, 633 3rd Ave, 3rd Floor, New York, NY, 10017, USA; Division of Epidemiology, Department of Medicine, Vanderbilt Epidemiology Center, Vanderbilt-Ingram Cancer Center, Vanderbilt University School of Medicine, 2525 West End Ave, Nashville, TN 37203, USA; Division of Epidemiology, Department of Medicine, Vanderbilt Epidemiology Center, Vanderbilt-Ingram Cancer Center, Vanderbilt University School of Medicine, 2525 West End Ave, Nashville, TN 37203, USA; Division of Epidemiology, Department of Medicine, Vanderbilt Epidemiology Center, Vanderbilt-Ingram Cancer Center, Vanderbilt University School of Medicine, 2525 West End Ave, Nashville, TN 37203, USA; Department of Biomedical Informatics, Vanderbilt University School of Medicine, 2525 West End Ave, Nashville, TN 37203, USA; Division of Epidemiology, Department of Medicine, Vanderbilt Epidemiology Center, Vanderbilt-Ingram Cancer Center, Vanderbilt University School of Medicine, 2525 West End Ave, Nashville, TN 37203, USA; Department of Biochemistry & Molecular Biology, University of Calgary, HMRB 231, 3330 Hospital Drive NW, Calgary, AB T2N 4N1, Canada; Department of Medical Genetics, University of Calgary, 3330 Hospital Drive NW, Calgary, AB T2N 4N2, Canada; Department of Mathematics & Statistics, University of Calgary, Mathematical Sciences 476, 2500 University Drive NW, Calgary, AB, T2N 1N4, Canada; Alberta Children's Hospital Research Institute, University of Calgary, Heritage Medical Research Building, 3330 Hospital Dr. NW, Calgary, AB T2N 4N1, Canada; Hotchkiss Brain Institute, University of Calgary, Health Research Innovation Centre, 3330 Hospital Drive NW, Calgary, Alberta, T2N 4N1, Canada; Division of Epidemiology, Department of Medicine, Vanderbilt Epidemiology Center, Vanderbilt-Ingram Cancer Center, Vanderbilt University School of Medicine, 2525 West End Ave, Nashville, TN 37203, USA; Department of Biomedical Informatics, Vanderbilt University School of Medicine, 2525 West End Ave, Nashville, TN 37203, USA

## Abstract

Transcriptome-wide association studies (TWAS) have been successful in identifying disease susceptibility genes by integrating *cis-*variants predicted gene expression with genome-wide association studies (GWAS) data. However, *trans*-variants for predicting gene expression remain largely unexplored. Here, we introduce transTF-TWAS, which incorporates transcription factor (TF)-linked *trans-*variants to enhance model building for TF downstream target genes. Using data from the Genotype-Tissue Expression project, we predict gene expression and alternative splicing and applied these prediction models to large GWAS datasets for breast, prostate, lung cancers and other diseases. We demonstrate that transTF-TWAS outperforms other existing TWAS approaches in both constructing gene expression prediction models and identifying disease-associated genes, as shown by simulations and real data analysis. Our transTF-TWAS approach significantly contributes to the discovery of disease risk genes. Findings from this study shed new light on several genetically driven key TF regulators and their associated TF–gene regulatory networks underlying disease susceptibility.

## Graphical Abstract

**Graphical Abstract:**
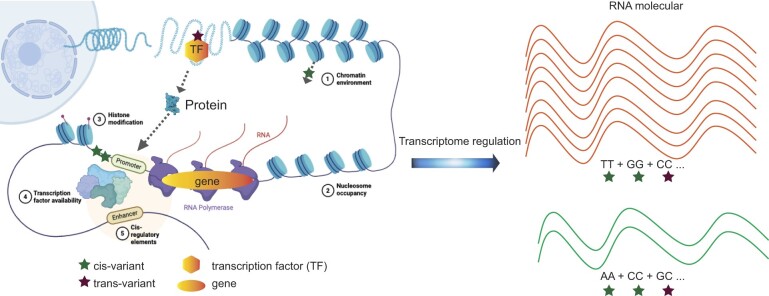

## Introduction

Approximately 90% of risk variants identified through genome-wide association studies (GWAS) are located in non-coding such as intergenic regions, which suggests that they may affect disease risk by dysregulating gene expression (1–4). Fine-mapping of genetic risk loci, along with functional experiments, provide strong evidence that regulatory variants in linkage disequilibrium (LD) with GWAS-identified risk variants disrupt DNA-binding affinities of specific transcription factors (TFs) and modulate the expression of susceptibility genes (2,5–8). Thus, identifying risk TFs, whose DNA bindings are altered by risk-associated genetic variations, and their controlling genes can greatly improve our understanding of transcriptional dysregulation in human diseases, including cancers (9–12). A pioneering study analyzed chromatin immunoprecipitation followed by sequencing (ChIP-seq) data for TFs such as FOXA1 in multiple breast cancer cell lines, along with GWAS-identified risk variants. The findings suggest that regulatory variants confer breast cancer risk by mediating altered FOXA1 binding affinities (13). Subsequent studies have revealed multiple breast cancer risk-associated TFs such as ESR1, MYC and KLF4 (14,15) through interrogating data on gene expression, TF ChIP-seq and GWAS-identified risk variants. We recently conducted a comprehensive analysis of TF ChIP-seq and GWAS data for breast cancer, and developed an analytical framework to identify TFs that contribute to breast cancer risk. Our study revealed that the genetic variations of 22 TFs were significantly associated with breast cancer risk and highlighted genetic variations of TF–DNA bindings (particularly for FOXA1) underlying breast cancer susceptibility (16).

Transcriptome-wide association studies (TWAS) have successfully uncovered large numbers of putative susceptibility genes for cancers and other diseases, and many of these genes have been further supported by functional experiments (17–20). In TWAS, a reference with both transcriptome and variant genotyping data from a small set of subjects, such as the Genotype-Tissue Expression project (GTEx), is used to build prediction models of gene expression for downstream association analyses. However, the accuracy of gene expression prediction models could be compromised if variants are located in non-regulatory elements. Additionally, prediction reliability can decrease even if the variants are situated in TF binding sites, while the corresponding TFs are not actively transcribed (21–24). Thus, it is essential to improve model building using transcribed TF-occupied putative regulatory variants to predict gene-expression, as shown in our recent approach, sTF-TWAS (25). Our approach focuses exclusively on *cis-*variants located in susceptible TF-occupied *cis-*regulatory elements (STFCREs), ranked by risk-associated TFs (16), for gene expression model construction. Our sTF-TWAS analysis using the above models and GWAS summary statistics can significantly enhance the detection of disease susceptibility genes, outperforming conventional TWAS methods (25).

Despite the progress made by TWAS in recent years (24–30), the current models for predicting gene expression were primarily based on *cis-*variants (<1 Mb distance), which generally account for only a modest proportion of disease heritability (31). In comparison, *trans-*variants may have more impact on the disease phenotype due to their advantages in population selection pressure, compensatory post-transcriptional buffering and gene expression regulation (32–34). It is estimated that 60%–90% of the heritability of gene expression can be explained by distal genetic variation in various tissues (34). However, including *trans-*variants in TWAS analysis is a challenge due to their overwhelming numbers on gene expression, compared to *cis-*variants, requiring larger sample sizes for detection (35). Recently developed approaches, BGW-TWAS and MOSTWAS, have integrated the analysis of *trans*-expression quantitative trait loci (*trans-*QTL) and demonstrated superior performance compared to S-PrediXcan and other methods (36,37). However, neither approach considered TF-based cell-type-specific regulatory elements to connect *trans-*variants with downstream target genes, despite the premise that risk TFs significantly contribute to risk loci and gene expression associated with cancer risk. Furthermore, these *trans-*eQTL approaches could potentially produce false positives for co-expression TF–genes due to their high sequence similarity and artificial mapping (38). Therefore, an integrative TF-based epigenetic data approach (i.e. TF ChIP-seq data) is needed to prioritize *trans-*variants that may play a regulatory role in the expression of downstream target genes.

In this work, we introduced transTF-TWAS, which included TF-linked *trans-*variants, together with *cis-*variants for prediction model building, in an effort to improve disease susceptible gene discovery. We showed that transTF-TWAS outperforms other methods by significantly improving gene expression prediction models and identifying disease risk genes. Specifically, we conducted transTF-TWAS to analyze both gene expression and alternative splicing with data generated from multiple normal tissues from the GTEx and large-scale GWAS data for breast, prostate and lung cancers, as well as three brain disorders, to identify disease susceptibility genes (Supplementary Data 1).

## Materials and methods

### Data resources

We obtained the individual-level genotype dataset from GTEx (v8) (39,40), which was quality controlled using PLINK (41). Summary statistics of GWAS data for breast cancer were obtained from the Breast Cancer Association Consortium, which has generated GWAS data for 122 977 cases and 105 974 controls from European descendants. GWAS data for prostate cancer were released from the European descendants of the Prostate Cancer Association Group to Investigate Cancer Associated Alterations in the Genome (42), with 79 194 cases and 61 112 controls from European descendants. GWAS data for lung cancer were obtained from the websites of the Transdisciplinary Research of Cancer in Lung of the International Lung Cancer Consortium (TRICL-ILCCO) and the Lung Cancer Cohort Consortium (LC3) (43), with 29 266 cases and 56 450 controls from European descendants. GWAS summary statistics for schizophrenia (SCZ, *N* = 70 100), Alzheimer’s disease (AD, *N* = 22 246) and autism spectrum disorder (ASD, *N* = 10 263) were downloaded from the Psychiatric Genomics Consortium website.

The TF-occupied regulatory variants for breast, prostate, lung cancers and three brain disorders were collected based on ChIP-seq data of TFs generated in disease related cell lines from the Cistrome database (44). We evaluated their quality control based on guidance from the database and selected high-quality datasets for downstream analysis. Detailed ChIP-seq data for breast, prostate, lung cancers and brain disorders were described in our previous work (16,25).

We included germline whole genome sequencing (WGS) and RNA-sequencing (RNA-seq) data from GTEx (release 8) (39,40) for normal breast tissue, prostate tissue, lung tissue and brain cortex tissue. We selected tissue samples from 151 women for breast tissue, 221 men for prostate tissue, 515 individuals for lung tissue and 205 individuals (both sexes) for brain cortex tissue. The fully processed, filtered and normalized gene expression data matrices (in BED format) were downloaded from the GTEx portal. The WGS file and sample attributes were obtained from dbGaP, and the subject phenotypes for sex and age information were obtained from the GTEx portal. The covariates used in eQTL analysis were obtained from GTEx_Analysis_v8_eQTL_covariates.tar.gz, and the covariates for sQTL analysis were obtained from GTEx_Analysis_v8_sQTL_covariates.tar.gz, both of which were downloaded from the GTEx portal. Normal breast tissue samples for both RNA-seq and genotyping from 181 individuals of European descent were collected through the Kome. Genotype and gene expression data generation and processing have been described in our previous sTF-TWAS work (25).

We downloaded approximately 3.6 million DNase I hypersensitive sites (DHSs) regions within the human genome sequence (45). The enhancer regions were downloaded from the EpiMap repository (46), which contains ∼2 M non-tissue specific enhancer regions. The CAGE peak regions were downloaded from FANTOM5 (47), and we also included all regions within the transcription start site (TSS) ±2 K for each gene as promoter regions. The eQTLs were downloaded from the GTEx portal (39,40) and eQTLGen (48). The Enhancer to gene link information across 833 cell-types was downloaded from the EpiMap repository (46). We all used cell-type specific chromatin–chromatin interaction data from the 4D genomics and previous literature (49,50).

To analyze cancer-related susceptibility genes, we downloaded a list of gene sets from the Molecular Signatures Database (MGB) on Gene Set Enrichment Analysis. Additionally, we downloaded lists of predisposition genes from previous literature (51,52), cancer-driven genes from two previous sources (53,54), and Cancer Gene Census (CGC) (55) from the COSMIC website. Cancer driver genes, or CGC, were identified by their significantly higher mutation frequencies in somatic mutation data compared to genome-wide controls, providing evidence of their role in the initiation and progression of cancer (e.g. oncogenes or tumor suppressors). To investigate the effect of an individual gene on essentiality for the proliferation and survival of cancer cells, we downloaded two comprehensive datasets, ‘sample_info.csv’ and ‘CRISPR_gene_effect.csv’, from DepMap Public 21Q4.

### Identifying *cis-*variants associated with TFs

To determine *cis-*variants that potentially regulate TF expression, we first prioritized putative regulatory variants by only including TF-occupied variants that were located in DHSs (45), enhancer regions (46) and promoter regions (46). Of them, the significant associations between a TF and its *cis-*variants were identified at a nominal *P* < 0.05, based on the eQTL analysis in both target tissues and whole blood samples using data from the GTEx portal (39,40) and eQTLGen (48). Furthermore, we also analyzed epigenetic data to search for regulating evidence by these variants through interactions with proximal promoters or distal enhancer-promoter regions. Specifically, we examined if these variants were located in the promoter region of a TF (TSS ± 2 K) or enhancer region, with evidence of the enhancer linking to the TF based on expression-enhancer activity correlation across 833 cell-types from the EpiMap repository (46), as well as chromatin–chromatin interaction data from the 4D genomics and previous literature (49,50). Finally, the *cis-*variants associated with TFs were identified based on the significant associations from eQTL results, and/or the regulatory evidence from the variants linked to the TF.

### Gene expression prediction model building based on *trans*-variants

We analyzed TF ChIP-seq data generated in target cancer-related cells to characterize their genome-wide binding sites for susceptible TFs using data from the Cistrome database (44). We next characterized each gene potentially regulated by all possible susceptible TFs based on the evidence of their TF–DNA binding sites located in its flanking 20 Kb of TSS (i.e. number of *G* TFs). For each TF, we assessed the performance of a prediction model that utilized its TF-linked *trans*-variants to predict expression of each target gene using the Group Lasso method. We trained a Group Lasso to select a group of TF-linked *trans*-variants from each TF (i.e. 1 to G TF).


\begin{equation*}{\boldsymbol{Loss\ }}\left( {{{{\boldsymbol{\beta }}}^{\boldsymbol{*}}}} \right) = {\boldsymbol{argmin}}\left| {\left| {{\boldsymbol{y}} - {\boldsymbol{X\beta }}} \right|} \right|_2^2 + {\boldsymbol{\ \lambda }}\mathop \sum \limits_{{\boldsymbol{g}} = 1}^{\boldsymbol{G}} ||{{{\boldsymbol{\beta }}}_{\boldsymbol{g}}}|{{|}_2}\end{equation*}

The coefficients in ${\boldsymbol{\beta }}$ are divided into *G* groups and ${{{\boldsymbol{\beta }}}_{\boldsymbol{g}}}$ denotes the coefficients vector of variants in the *g*th group. ***X*** are all *trans*-variants from *G* groups. ${\boldsymbol{y}}$ is normalized gene expression data generated in different tissue samples from GTEx v8. In Group Lasso, the regularizor, $||{{{\boldsymbol{\beta }}}_{\boldsymbol{g}}}|{{|}_2}$, also called ${{l}_{2,1}}$-norm, consists of the intra-group non-sparsity via ${{l}_2}$-norm and inter-group sparsity via ${{l}_1}$-norm. Only significant models were used to determine those groups of TF-linked *trans*-variants that may affect the expression of the gene. The final set of TF-linked *trans*-variants was identified for downstream gene expression model building by combining the groups from the significant models. We next built gene expression prediction models for the final sets of TF-linked *trans*-variants and *cis-*variants using standard Elastic Net under our sTF-TWAS framework. For each gene, the gene expression level was regressed on the number of effect alleles (0, 1 or 2) for each genetic variant, with adjustment for the top five genotyping PCs, age and other potential confounding factors (PEERs). We used 30 PEER factors for our downstream model building based on the recommendation for breast, prostate and brain tissues, and 60 PEER factors for lung tissue. Prediction model performance was assessed using the R^2^ via a 10-fold cross-validation.

### Simulation study and external verification to assess gene expression predictions

To evaluate prediction performance of our developed approach, we simulated scenarios for each gene that had the equal number of artificial TF groups with our transTF-TWAS, termed transTF-TWAS (R). We randomly generated the same number of *trans-*variants (> 1 Mb distance) within each TF group with our transTF-TWAS. Similarly, we next used Group Lasso to select significant groups from the artificial TF groups. The final set of *trans*-variants was identified for downstream gene expression model building by combining the groups from the significant models. We next built gene expression prediction models for the final sets of *cis* TF-occupied variants under sTF-TWAS framework. The models of genetically predicted gene expression were built in breast normal tissue from the GTEx project. To externally verify gene expression prediction performance, we first used the same analytical protocol to build the prediction models using standard Elastic Net based on normalized gene expression data generated in breast tissue from the GTEx (v8), and then we re-calculated the prediction performances in terms of variance explained (R^2^) using selected variants trained from the GTEx based on an independent dataset generated in breast normal tissue from Kome, where the genotype and gene expression data were processed following the protocol in GTEx.

### Association analyses between predicted gene expression and cancer risk

To evaluate associations of genetic predicted gene expression with cancer risk, we applied the weight matrix obtained from the gene prediction models to the summary statistics implemented in S-PrediXcan (56). The statistical method described in the following equation, also described elsewhere (57,58), was used for association analyses.


\begin{equation*}{{Z}_g} \approx \ \mathop \sum \limits_{l \in {\mathrm{Mode}}{{{\mathrm{l}}}_g}} {{w}_{lg}}\frac{{{{{\hat{\sigma }}}_l}}}{{{{{\hat{\sigma }}}_g}}}\ \frac{{{{{\hat{\beta }}}_l}}}{{{\mathrm{se}}\ ({{{\hat{\beta }}}_l})}}\end{equation*}

Here, Z-score was used to estimate the association between predicted gene expression and cancer risk. Here, ${{w}_{lg}}$ is the weight of genetic variant $l$ for predicting the expression of gene $g$. ${{\hat{\beta }}_l}$ and ${\mathrm{se}}\ ({{\hat{\beta }}_l})$ are the GWAS-reported regression coefficients, and its standard error for variant $l$, and ${{\hat{\sigma }}_l}$ and$\ {{\hat{\sigma }}_g}$ are the estimated variances of variant $l$ and the predicted expression of gene $g,$ respectively.

By comparison, we also performed TWAS analysis using PUMICE (30) (Prediction Using Models Informed by Chromatin conformations and Epigenomics) with default settings. PUMICE improves the accuracy of transcriptomic imputation through utilizing tissue-specific 3D genomic and epigenomic data to prioritize regions that harbor *cis-*regulatory variants. The source codes of PUMICE were obtained from https://github.com/ckhunsr1/PUMICE. The precomputed models trained in breast, prostate and lung tissue from GTEx v8 can be found under https://github.com/ckhunsr1/PUMICE/tree/master/models_GTEx_v8.

### Simulation study to assess type-I error

We conducted the null simulation to evaluate the type-I error of our transTF-TWAS. The individual-level genotype data provided by the GTEx was used as the reference dataset to simulate the gene expressions and the 1000 Genomes Project dataset was used to simulate phenotype and conduct GWAS/TWAS analyses. We first randomly generated phenotype values (0 or 1) independently from the genotype. We then conducted logistic regression analysis to generate the GWAS summary statistics using the phenotype values and the genotype data from the 1000 Genomes Project. We next randomly generated putative TF-occupied regulatory variants and TF-linked *trans*-variants for model training. The protocol for randomly selecting these putative TF-occupied regulatory variants was the same as our previous sTF-TWAS work (25). Specifically, to generate 50 K *cis-*variants occupied by an artificial TF, we prioritized a set of variants using generalized linear mixed models to analyze GWAS summary statistics of all genetic variants and the TF’s binding status. Specifically, we randomly assigned 50 K TF-occupied variants to a value ‘1’ and the remaining variants to a value ‘0’ (i.e. 1 for a variant located in a TF binding site, 0 otherwise). We then used generalized linear mixed models to estimate an association between the Chi-squared values (Y) and TF binding status of genetic variants. We prioritized a set of variants based on the association for a given ‘TF’ with cancer risk at *P* < 0.05. We repeated the above statistical analysis (>4000 times) and used prioritized sets of variants as TF-occupied *cis-*regulatory variants together with the randomly selected *trans*-variants for our downstream model training. To generate a set of TF-linked *trans*-variants, we randomly selected 50 *cis-*variants as eQTL variants for an artificial TF. We repeated the process to generate 10 sets of *cis-*variants for 10 artificial TFs. Theses TFs are all considered as TF–gene pairs for downstream model training. Group Lasso was used to train each set of *cis-*variants for each TF in the gene expression prediction model to examine if the TF significantly contributed to the prediction (i.e. non-zero coefficients). For the groups surviving regularization, they were considered to be *trans*-variants, along with TF-occupied *cis-*regulatory variants, and were all included in the Elastic Net to train and prioritize genetic variants to predict gene expression. We further conducted TWAS analysis based on the well predicted gene expression models (R^2^ > 0.01) and GWAS summary statistics. We repeated the above simulation processes 100 times to increase the robustness.

### Simulation study to assess statistical power

The process of simulation is structured into three components: (1) high-level genetic architectures, i.e. causality and horizontal pleiotropy; (5) trait heritability and expression heritability; and (6) expression heritability contributed by *cis-* and *trans*-variants. We conducted simulations under two representative high-level architectures: (1) causality where genotype causes phenotypic changes via the mediation of gene expression, and (5) horizontal pleiotropy where genotype contributes to phenotype and gene expression independently. To simplify simulations under both scenarios, we simulated gene expressions and phenotypes using an additive genetic architecture. Under the additive architecture, phenotypes and gene expressions are simulated by the sum of genetic effects:


\begin{equation*}f\left( X \right) = {\mathrm{\ }}\mathop \sum \limits_{i = 1}^n {{\beta }_i}{{x}_i} , \end{equation*}

where $X$ is the genotype matrix from either the GTEx or 1000 Genomes Project, $X$ = {${{x}_1},{{x}_2}$,…,${{x}_n}$}. The effect size ${{\beta }_i}$ is drawn from the standard normal distribution N (0,1), which will be used in the downstream TWAS analysis.

The formula for simulating gene expression is


\begin{equation*}{{z}^{\left( 1 \right)}} = f\left( {{{X}^{\left( 1 \right)}}} \right) + {\mathrm{\ }}\varepsilon \end{equation*}

\begin{equation*}{{z}^{\left( 2 \right)}} = f\left( {{{X}^{\left( 2 \right)}}} \right) + {\mathrm{\ }}\varepsilon , \end{equation*}

where ${{z}^{( 1 )}}$ is the gene expression simulated using genotype from the GTEx (${{X}^{( 1 )}})$, and ${{z}^{( 2 )}}$ is the gene expression simulated using genotype from the 1000 Genomes Project (${{X}^{( 2 )}}$). Here, the super-index (1) indicates that the data is from the expression dataset GTEx, whereas the super-index (5) indicates the data is from the GWAS dataset, which is the 1000 Genomes Project in this simulation. The formula for simulating phenotype under the causality scenario is


\begin{equation*}{{y}^{\left( 2 \right)}} = {{g}_c}\left( {{{z}^{\left( 2 \right)}},{{X}^{\left( 2 \right)}}} \right) + \varepsilon , \end{equation*}

where ${{y}^{( 2 )}}$ is the phenotype simulated with ${{g}_c}$ function, and ${{g}_c}( {{{z}^{( 2 )}},{{X}^{( 2 )}}} )$ = ${{z}^{( 2 )}}{\mathrm{\ }}$+ $\varepsilon$.

The formula for simulating phenotype under the pleiotropy scenario is


\begin{equation*}{{y}^{\left( 2 \right)}} = {{g}_p}\left( {{{X}^{\left( 2 \right)}}} \right) + \varepsilon , \end{equation*}

where ${{y}^{( 2 )}}$ is the phenotype simulated with ${{g}_p}$function, and ${{g}_p}( {{{X}^{( 2 )}}} )$ employs the same format to $f{\mathrm{\ }}( {{{X}^{( 2 )}}} )$, except that the variance component is rescaled by gene expression heritability instead of trait heritability. The above genetic architectures define how genetic components contribute to each phenotype. Using the genetic components, we generated phenotypes where the variance component attributed to genotype, or heritability, equals the preselected value h^2^. That is, given the variance of the phenotype's genetic component as $\sigma _g^2$, we solved $\sigma _e^2$ to satisfy that $\sigma _g^2$/ ($\sigma _g^2$ + $\sigma _e^2$) = h^2^. We then sampled from the normal distribution N (0,${\mathrm{\ }}\sigma _e^2$) to determine the strength with which non-genetic components such as noise or environmental effects contribute to phenotype. Finally, the sum of the genetic and non-genetic components were used as the simulated phenotype in association mapping and power calculations.

To simulate the effect of TF-occupied *cis-*regulatory variants, we followed the same procedure as we did for sTF-TWAS. For each target gene, we first randomly selected 200 variants from the local gene regions (± 1 Mb of the gene body) as potential predictors. When simulating gene expression heritability contributed by trans-variants, we randomly selected 5 (or 10) genetic variants with minor allele frequency larger than 1% from these 200 variants as the functional causal *cis-*located genetic variants. In addition, we simulated the effect of *trans*-located genetic variants. We first randomly selected 10 regions across the whole genome (> 1 Mb of the gene body), with each region containing 50 randomly selected genetic variants as 10 *trans-*variants group. Among these 10 regions, we randomly select 3 (or 4) regions as the real functional *trans-* region, and among each *trans-* region, we randomly select 5 (or 10) genetic variants as the real causal *trans-*located genetic variants. In total, we simulated 20 (or 50) real causal genetic variants for each target gene. The relative variance components contributed between *cis-* and (aggregated) *trans-* genetic variants were then re-scaled to the desired ratio using a similar re-scaling model that structures the relative contribution between genetic contribution ($\sigma _g^2$) and noise ($\sigma _e^2$) described above.

To illustrate the improved statistical power of transTF-TWAS, which incorporates prior knowledge of the potential *trans-*variants group, we compared the statistical power of transTF-TWAS with two other TWAS approaches using only *cis-*variants. First, PrediXcan: using all *cis-*variants (±1 Mb of the gene body); second, sTF-TWAS: using 200 randomly selected genetic variants; and third, one TWAS approach also including *trans*-genetic variants for predictive model building, BGW-TWAS: using all genetic variants across the whole genome.

For all models, the gene expressions were simulated using genotype data from GTEx, and phenotypes were simulated using genotypes from the 1000 Genomes Project. This simulated situations where the real gene expressions were not available in the target dataset (represented by the 1000 Genomes Project genotype and simulated phenotype); instead, we used another reference dataset (represented by the GTEx genotype and simulated expressions) to train the weights for each gene. For each of the genetic architectures and their associated parameters, we simulated 1000 datasets, in which causal variants were randomly selected. We then tested each protocol’s ability to successfully identify the genes in each dataset, where success was defined as a Bonferroni-corrected *P*-value that is lower than a predetermined critical value (0.05).

### Genetically driven key TF regulators and their associated TF–gene networks regulating breast cancer susceptibility genes

For each of the identified putative susceptibility genes, we evaluated the lead variant that presented the strongest associations with cancer risk in the prediction model. If the lead variant was a *trans*-located variant, we next identified its potential regulated TF based on the previous analysis of the TF-*cis-*regulatory-variant (see the preceding section). A TF–gene pair was further determined based on the above information from the lead *trans-*located variant linked to both the gene and TF. Based on the information from TF–gene pairs, a TF-transcriptional network was built using Cytoscape 3.9.1 (59). To determine whether our identified susceptibility genes showed significant enrichment in a TF (acting as an up-regulator based on TF–gene pairs) of interest, we compared this TF to the remaining combined TFs as background using Fisher’s exact test. This statistical test employs a 2 × 2 Data to ascertain the significance of enrichment for the TF of interest.

### Sp-transTF-TWAS analysis

In our transTF-TWAS, we incorporated both putative *cis-*regulatory variants located in STFCRES and TF-linked translocated variants for gene expression prediction and downstream association analysis. As an extension of transTF-TWAS with a broader genetic regulatory mechanism (e.g. eQTL versus alternative splicing QTL), we also utilized both putative *cis-*regulatory variants in STFCRES and TF-linked translocated variants for predicting alternative splicing expression. For those genetically well-predicted alternative splicing (R^2^ > 0.01), we applied these prediction models to GWAS to assess the association between genetically predicted alternative splicing and cancer risk.

### Annotation of the identified genes using cancer-related gene database

To verify the evidence of whether the TWAS-identified genes are related to cancer susceptibility, we extracted cancer related gene sets from the MGB database. Putative cancer related genes were characterized based on their annotation with the key words ‘breast cancer’, ‘prostate cancer’ and ‘lung cancer’. We calculated the number and percentage (success rate) of putative cancer related genes that overlapped with those extracted from the MGB database among the identified genes in this study. Previous TWAS or eQTL studies for breast cancer (7,16,25,57,60), prostate cancer (3,25,61–63) and lung cancer (3,25,64) reported genes related with these cancers. Genetic variants related with the risk of breast cancer (2,65), prostate cancer (66) and lung cancer (43,67) were reported in previous GWAS. We also examined the overlap between the genes identified in this study with predisposition genes, cancer driver genes and CGC-based gene sets. To evaluate whether our identified genes were significantly enriched in these cancer-related genes, we conducted enrichment analysis based on the probability mass function of the hypergeometric distribution. Similar to our previous work (25), the *P*-value was calculated as phyper function implemented in R.

### Pathway enrichment analysis and effect of gene silencing on cell proliferation

The IPA tool was used to evaluate the functional enrichment of canonical biological pathways for the identified putative cancer susceptibility genes in breast, prostate and lung cancers. CRISPR-Cas9 has enabled genome-scale identification of genes that are important for the proliferation and survival of cancer cells, which have been widely used for genetic studies (16,68,69). The CERES score, a widely used metric derived from CRISPR-Cas9 essentiality screens, provides an estimation of gene dependency levels (68). We obtained the CERES score for each gene in a given cell from the DepMap portal. Subsequently, we calculated the median negative CERES score across 45 breast-relevant cells, 8 prostate-relevant cells and 130 lung-relevant cells. A CERES value below –0.5 serves as the cutoff to denote the essentiality of a gene for cell proliferation in CRISPR-Cas9 gene silencing experiments (68,70).

The probability mass function of the hypergeometric distribution is: $P{\mathrm{\ }}( x )$ = $$\frac{{\left( {\begin{array}{@{}*{1}{c}@{}} m\\ x \end{array}} \right){\mathrm{*\ }}\left( {\begin{array}{@{}*{1}{c}@{}} n\\ {k - x} \end{array}} \right)}}{{{\mathrm{\ }}\left( {\begin{array}{@{}*{1}{c}@{}} N\\ k \end{array}} \right)}}$$, where m is the total number of genes in all cancer-related gene databases, which includes all predisposition genes, cancer drivers and CGC genes; *n* is the number of genes that are not included in the cancer-related gene databases (*n* = *N* – m, *N* = 19, 291 protein-coding genes based on the annotation from the Gencode.v26.GRCh38).

## Results

### Overview of transTF-TWAS framework

We introduced our new approach, transTF-TWAS, to build gene expression prediction models by adding TF-linked *trans*-variants together with *cis-*variants located in STFCREs under our previous sTF-TWAS framework. Specifically, we outlined several main steps below for identifying and prioritizing *trans*-variants to enhance gene expression predictions. We illustrated this methodology with the TF FOXA1 in breast cancer as an example.

**Figure 1.**
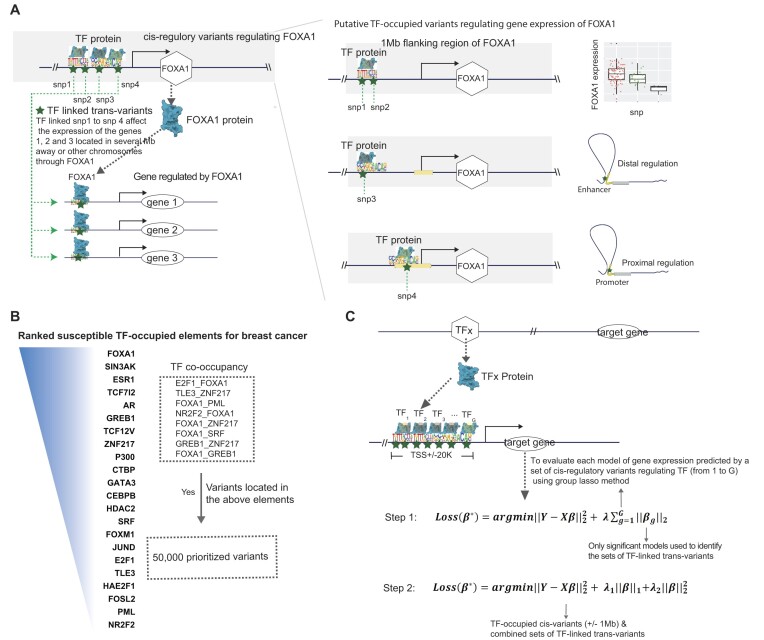
Overview of the Developed Analytical Framework. (**A**) An illustration of how to prioritize TF-linked *trans-*variants for prediction model building (using FOXA1 as an example). In the left panel, TF-linked *trans-*variants, such as snp1 to snp4, can influence the expression of TF-regulated genes located several megabases away or even on different chromosomes. The blue star represents a snp, while the blue irregular shape represents a TF protein that can occupy the genomic region where the snp is located. The right panel illustrates the identification of *cis-*variants associated with FOXA1 (Step I). snp1 and snp2 are identified as *cis-*eQTLs within a 1 Mb flanking region of the FOXA1 gene. snp3, located in a distant regulatory element, is shown to regulate the gene’s expression through distal enhancer-promoter interactions, as revealed by epigenetic data (e.g. chromatin–chromatin interactions). For snp4, epigenetic and location-based analyses can determine whether the variant resides in the gene’s proximal promoter. (**B**) A flow chart shows the prioritization of TF-occupied regulatory variants (50 K), ranked based on established TF-occupied elements associated with breast cancer risk. (**C**) The two-step gene expression prediction model in transTF-TWAS is illustrated. The top panel represents TF–gene pair discovery (Step II). In Step III, we assessed the performance of a prediction model that utilized its TF linked *trans-*variants to predict expression of each target gene using the Group Lasso method (i.e. number of G TFs; Figure 1C; see the ‘Materials and methods’ section). Under our sTF-TWAS framework, we next included the prioritized TF-linked *trans-*variants, together with the *cis-*variants, to build gene expression prediction models.

- Step I: Identifying *cis-*variants associate with TFs. We first identified *cis-* variants located in STFCREs that could potentially affect expression of a TF (e.g. FOXA1) by conducting *cis-*eQTL analysis and analyzing epigenetic data generated in breast-related cells. A set of the *cis-*variants regulating TF expression was determined based on the significant associations between TF gene expression and *cis-*variants (eQTL analysis), and/or variants with evidence of regulatory interactions with proximal promoters or distal enhancer-promoter regions (Figure 1A; see the ‘Materials and methods’ section).
- Step II: TF–gene pair discovery. We analyzed TF ChIP-seq data generated in breast cancer-related cells to characterize their genome-wide binding sites for susceptible TFs that have been identified in breast cancer from our prior work (16) (Figure 1B; see the ‘Materials and methods’ section). We next characterized each gene potentially regulated by all possible susceptible TFs based on the evidence of the TF–DNA binding sites that are located in its flanking of TSS (±20 K; Figure 1C; see the ‘Materials and methods’ section). *Cis-*variants associated with TFs (subsequently termed TF-linked *trans*-variants) have the potential to regulate TF protein expression, which may consequently alter the gene expression of downstream targets. As a result, TF-linked *trans*-variants can influence the expression of genes located several megabases away or even on different chromosomes.
- Step III: Model training and disease-trait association analysis. For each TF, we assessed the performance of a prediction model that utilized its TF linked *trans*-variants to predict expression of each target gene using the Group Lasso method (i.e. number of G TFs; Figure 1C; see the ‘Materials and methods’ section). The Group Lasso’s property of encouraging between-group sparsity and within-group retainment aligns to our intention of selecting the actual functioning TFs and then retaining their *cis-*variants. The groups that survive the regularization correspond to those of TF-linked *trans*-variants that may affect the expression of the gene. The final set of TF-linked *trans*-variants was identified for downstream gene expression model building by combining the groups from the significant models using Elastic Net (Figure 1C and Supplementary Data 1; see the ‘Materials and methods’ section). Under our sTF-TWAS framework, we next included the TF-linked *trans*-variants, together with the prioritized *cis-*variants, to build gene expression prediction models. Here, we only focused on the set with 50 K *cis-*variants (Figure 1B), as the identified genes were highly overlapped among analyses with different numbers of variants (i.e. 50 K versus 500 K variants) in our prior work (16,25). We conducted TWAS analyses by applying the gene expression prediction models, respectively, to GWAS summary statistics for breast, prostate and lung cancers and other diseases to search for their susceptibility genes (Supplementary Data 1).

### Simulation study

**Figure 2.**
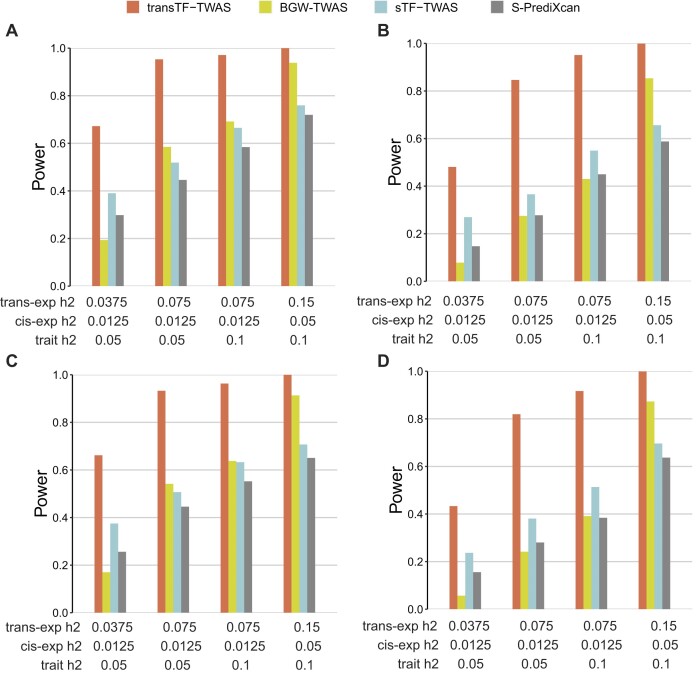
Power comparison under pleiotropy and causality scenarios for gene expression heritability that are substantially contributed by TF-linked *trans*-variants. Power is indicated on the y-axis. All panels are results under an additive genetic architecture, with differing *trans-*variants expression heritability, *cis-* variants expression heritability and trait heritability denoted below each panel. (**A, B**) Under pleiotropy scenario. (**A**) A total of 20 causal genetic variants. (**B**) A total of 50 causal genetic variants. (**C,D**) Under causality scenario. (**C**) A total of 20 causal genetic variants. (**D**) A total of 50 causal genetic variants.

We conducted simulations to assess the enhancement of gene expression prediction models in statistical power by utilizing selected TF-linked translocated variants and *cis-*variants located in STFCREs. We compared with three existing TWAS methods (BGW-TWAS, sTF-TWAS, and S-PrediXcan), using either all *cis-*variants or randomly selected variants of equivalent numbers (see the ‘Materials and methods’ section). Initially, we validated that the type-I error of transTF-TWAS remained properly controlled, aligning with the null expectation, at a Bonferroni-corrected *P* < 0.05 (Supplementary Figure S1). Subsequently, we examined two scenarios: causality, where genotypes influence a phenotype through gene expression intermediaries, and horizontal pleiotropy, where genotypes influence a phenotype and expression independently (see the ‘Materials and methods’ section). In both scenarios, we observed that transTF-TWAS outperformed other TWAS methods when gene expression heritability was significantly contributed by *trans-*variants, while it exhibited comparable performance to sTF-TWAS when gene expression heritability was weakly influenced by *trans*-variants (Figure 2 and Supplementary Figure S2). Overall, the statistical power of transTF-TWAS increased proportionally with the heritability of both gene expression and phenotype traits, and decreased with an increased number of causal variants (Figure 2 and Supplementary Figure S2). These observations offer compelling evidence that our transTF-TWAS approach, integrating TF-linked *trans-*variants, holds superior statistical power compared to these earlier methods.

### Independent verification of gene expression prediction

To assess the performance of transTF-TWAS, we conducted simulations within an extension of our sTF-TWAS framework (25), termed transTF-TWAS (R), by incorporating randomly selected *trans*-variants of equal quantity (see the ‘Materials and methods’ section). Initially, we compared the predictive performance of gene expression models derived from transTF-TWAS with those from transTF-TWAS (R) and sTF-TWAS. In our analysis utilizing breast tissue data from GTEx, we observed that transTF-TWAS predicted slightly more genes than transTF-TWAS (R), with an R^2^ threshold > 0.01, while it predicted over 1800 genes more than sTF-TWAS (Supplementary Figure S3A). Moreover, employing independent datasets generated from normal breast tissue from the Susan G. Komen Normal Tissue Bank (Kome) (*n* = 181; see the ‘Materials and methods’ section), we demonstrated that a higher proportion of genes predicted by transTF-TWAS were validated, compared to the two alternative methods (Supplementary Figure S3B). Furthermore, by applying the prediction models to GWAS data in breast cancer, we identified 141 putative susceptibility genes using transTF-TWAS at a Bonferroni-corrected significance level of *P* < 0.05, exceeding the number identified by sTF-TWAS (62 genes) and transTF-TWAS (R) (41 genes) (Supplementary Figure S3C).

### TransTF-TWAS outperforms existing TWAS approaches in real data analysis

We next expanded our comparisons for transTF-TWAS with existing approaches including sTF-TWAS (25), PUMICE (30) and S-PrediXcan (71), in the analysis for breast, prostate and lung cancers. We showed that transTF-TWAS identified more genes than sTF-TWAS under multiple *P*-value cutoffs (Supplementary Figure S4A). As described above, we identified 141 putative susceptibility genes from transTF-TWAS, at a Bonferroni-corrected *P* < 0.05, while fewer genes were identified by sTF-TWAS (*n* = 62), S-PrediXcan (*n* = 52) and PUMICE (*n* = 42) (Supplementary Figures S5 and S6, and Supplementary Data 2). We conducted similar comparisons for prostate and lung cancers and demonstrated consistent trends of more genes identified by transTF-TWAS compared to the other three approaches (Supplementary Figures S5 and S6, and Supplementary Data 3 and 4).

Next, we utilized knowledge-based disease-relevant genes to evaluate the potential false positives of our gene-trait associations identified from transTF-TWAS, compared to other approaches. We conducted functional annotation for the genes identified by transTF-TWAS and sTF-TWAS with known target cancer-related genes of interest (see the ‘Materials and methods’ section). Our analysis revealed that transTF-TWAS detected more breast cancer-related genes (*n* = 61) compared to sTF-TWAS (*n* = 26) (Supplementary Figure S4B), with a comparable proportion (62.2% for transTF-TWAS versus 61.9% for sTF-TWAS) (Supplementary Figure S4C). Additionally, our approach identified an overall higher quantity and a higher or comparable proportion of known cancer-related genes for both prostate and lung cancers, compared to other approaches (Supplementary Figure S4B and C).

### Genetically driven key regulators and their associated networks underlying cancer risk

**Figure 3.**
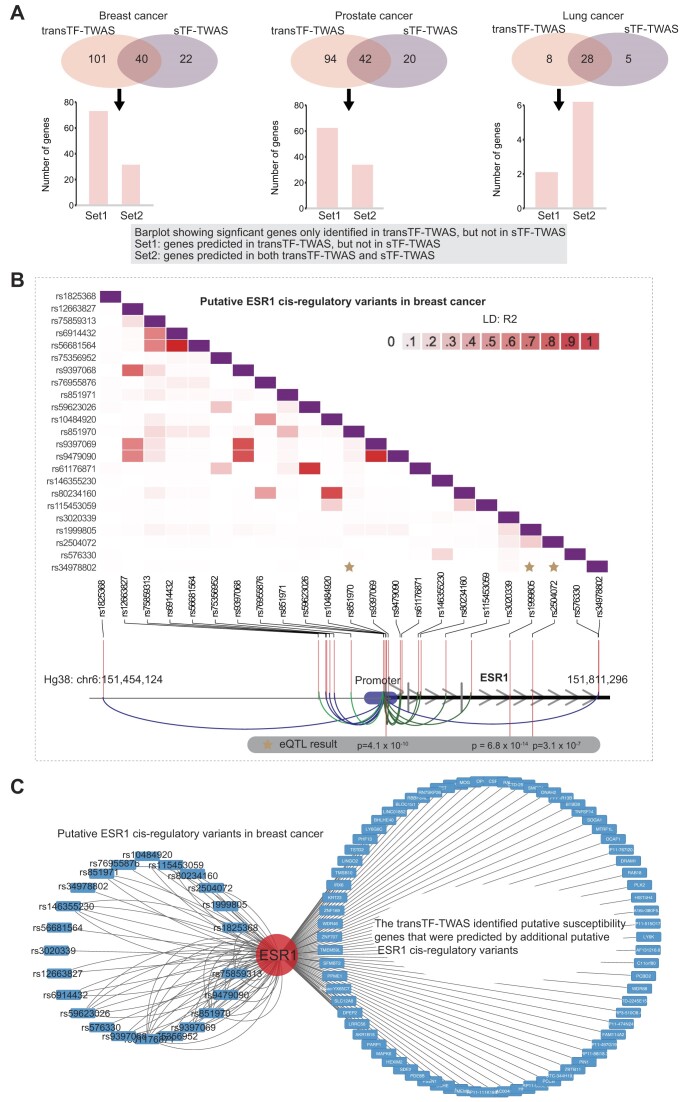
The gene regulatory network underlying cancer risk driven by master regulators. (**A**) Venn diagrams showing the number of putative susceptibility genes commonly or uniquely identified by transTF-TWAS and sTF-TWAS. An arrow points from the uniquely identified genes by transTF-TWAS to a bar chart showing: Set 1: Genes predicted in transTF-TWAS, but not in sTF-TWAS. Set 2: Genes predicted in both transTF-TWAS and sTF-TWAS. (**B**) A heatmap showing the LD structure among putative ESR1 *cis-*regulatory variants in breast cancer. The ESR1 *cis-*regulatory variants are *trans-*variants that present the strongest associations with cancer risk in the prediction model. (**C**) A network showing the connections between the putative ESR1 *cis-*regulatory variants in breast cancer and putative susceptibility genes identified by transTF-TWAS that were contributed by putative ESR1 *cis-*regulatory variants.

We showed that transTF-TWAS detected more genes than sTF-TWAS in breast, prostate and lung cancers; a large number of significant genes were uniquely detected by transTF-TWAS (Figure 3A). To further illustrate how these unique genes are contributed by *trans-*variants, we examined whether these genes can be predictable by sTF-TWAS. We found that most of the unique genes in breast and prostate cancers failed to be predicted by sTF-TWAS, indicating that the *trans-*variants significantly contributed to risk gene discovery via improved gene expression prediction performance (Figure 3A and Supplementary Data 5–7).

Of the identified genes, we next evaluated the lead *trans-*variants that presented the strongest associations with cancer risk in the prediction model for each of our identified putative susceptibility genes. In breast cancer, we observed that the lead variants were significantly enriched in the TFs ESR1 (*n* = 73 genes), followed by TCF7L2 (*n* = 13) and FOXA1 (*n* = 11) (Fisher’s exact test, *P* < 0.01 for all; Figure 3B and C, and Supplementary Data 8 and 9). In prostate cancer, we observed that the lead variants are significantly enriched in NKX3-1 (*n* = 61 genes), followed by GATA2 (*n* = 13) (Fisher's exact test, *P* < 0.01 for all; Supplementary Data 8 and 10). These results highlighted these genetically driven key TF regulators and their associated TF–gene regulatory networks that underlie cancer susceptibility.

**Figure 4.**
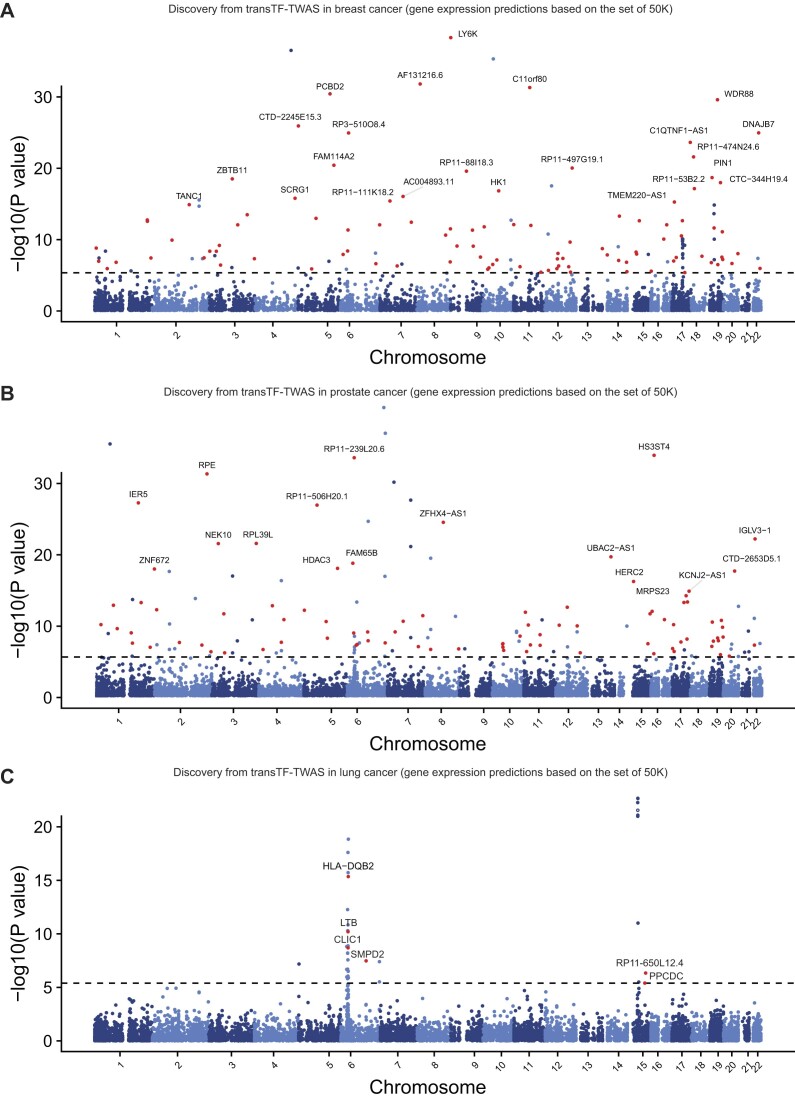
Putative susceptibility genes identified by transTF-TWAS. Manhattan plots showing the associations identified from transTF-TWAS. Red dots indicate all newly identified susceptibility genes, and the gray dashed line refers to Bonferroni-corrected *P* < 0.05. The newly identified putative susceptibility genes with *P* < 10^−15^ were highlighted with their names indicated. The *P*-values are the raw *P*-values from the Z-score test from TWAS (two-sided). (**A**) Breast cancer. (**B**) Prostate cancer. (**C**) Lung cancer.

To further characterize putative susceptibility genes and loci identified under our transTF-TWAS framework (Figure 4), we additionally analyzed alternative splicing (sp-transTF-TWAS; see the ‘Materials and methods’ section) for breast, prostate and lung cancers (Supplementary Figure S7 and Supplementary Data 11–13). We comprehensively compared our findings from both transTF-TWAS and sp-transTF-TWAS, with those reported from previous TWAS, eQTL or other genetic studies for breast (2,7,16,25,57,60,65), prostate (3,25,61–63) and lung cancers (3,25,64).

**Figure 5.**
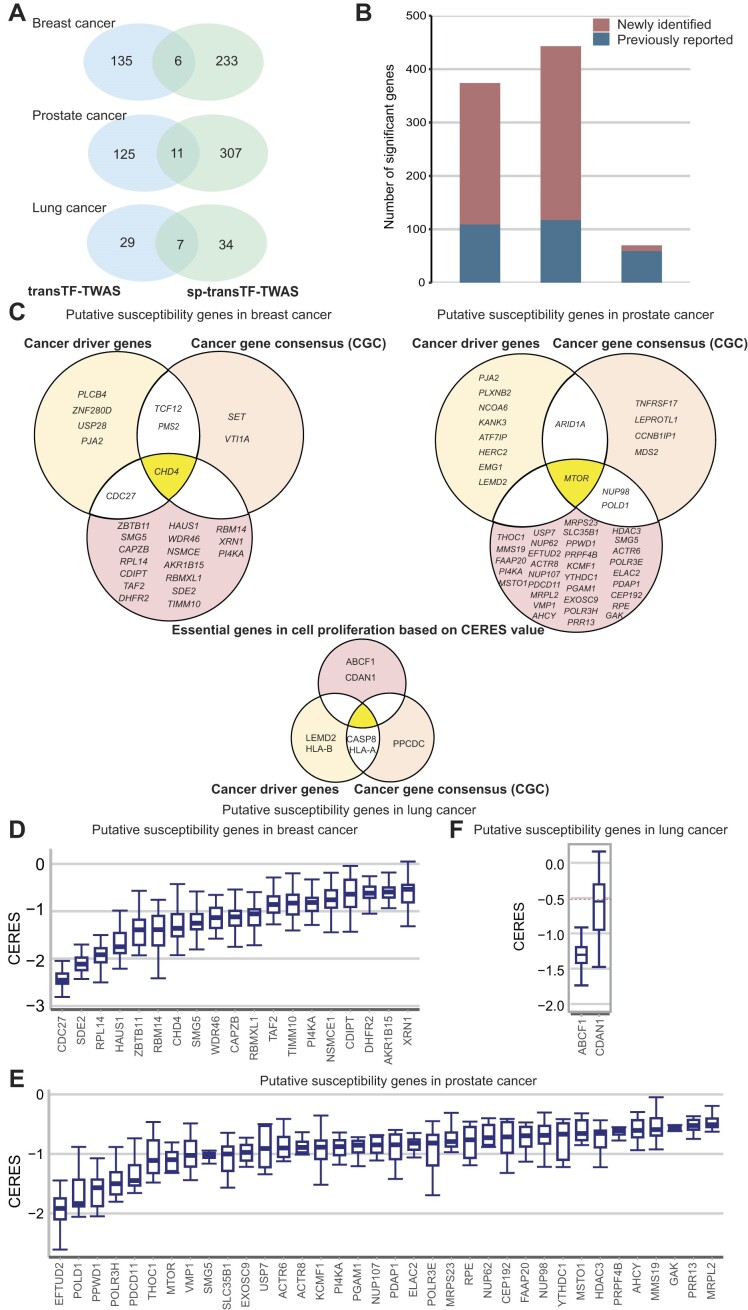
Putative susceptibility genes identified by transTF-TWAS and sp-transTF-TWAS. (**A**) Venn diagrams showing the number of putative susceptibility genes commonly or uniquely identified by transTF-TWAS and sp-transTF-TWAS. (**B**) Bar chart showing the total identified putative susceptibility genes combined from transTF-TWAS and sp-transTF-TWAS for breast, prostate and lung cancer. (**C**) Venn diagrams showing all newly identified genes that were cancer driven genes, CGC or genes with CERES←0.5 for breast cancer, prostate and lung cancer. (**D–F**) Boxplot showing all newly identified genes with evidence of essential roles in cell proliferation, based on a cutoff of median CERES values <–0.5 for (**D**) breast cancer (sample size: 45 cell lines), (**E**) lung cancer (sample size: 130 cell lines) and (**F**) prostate cancer (sample size: eight cell lines). In the boxplots shown in these figures, the whiskers denote the range; the boxes denote the interquartile range; the middle bars denote the median.

In our analysis, we investigated more alternative splicing than gene expression during model building due to the nature of the input data, resulting in the detection of more significant genes through sp-transTF-TWAS compared to transTF-TWAS. For breast cancer, we identified 141 putative susceptibility genes from transTF-TWAS and 239 putative susceptibility genes from sp-transTF-TWAS, at a Bonferroni-corrected *P* < 0.05. Combing the results from both analyses, we identified 374 putative breast cancer susceptibility genes, including 212 genes at 163 novel loci (>1 Mb away from any previous GWAS-identified risk variant for breast cancer), and 53 previously unreported, located in GWAS loci (Figure 5A and B, and Supplementary Data 14). For prostate cancer, we identified 136 putative susceptibility genes from transTF-TWAS and 318 putative susceptibility genes from sp-transTF-TWAS. Combining the results from both analyses, we identified 443 putative prostate cancer susceptibility genes, including 251 genes at 193 novel loci and 75 genes previously unreported that were located in GWAS loci (Figure 5A and B, and Supplementary Data 15). For lung cancer, we identified 36 putative susceptibility genes from transTF-TWAS and 41 putative susceptibility genes from sp-transTF-TWAS. Combining the results from both analyses, we identified 70 putative lung cancer susceptibility genes, including 2 genes at one novel locus and 9 genes previously unreported, located in GWAS loci (Figure 5A and B, and Supplementary Data 16). Taken together, our analysis revealed a total of 887 putative susceptibility genes for these three cancer types, including 137 that were previously unreported in GWAS loci and 465 in loci unreported by GWAS (Supplementary Data 17 and 18).

### Functional evidence of oncogenic roles for the identified putative susceptibility genes

Functional enrichment analyses using the Ingenuity Pathway Analysis (IPA) tool revealed that the identified putative susceptibility genes were significantly enriched in cancer-related pathways (*P* < 0.05; Supplementary Data 19; see the ‘Materials and methods’ section). Among the top enriched canonical pathways, we identified Intra-Golgi and Retrograde Golgi-to-ER Traffic, as well as DNA Methylation and Transcriptional Repression signaling for breast cancer; Cytosolic Iron-Sulfur Cluster Assembly and Glycerophospholipid Biosynthesis for prostate cancer; and cancer immune-related pathways such as Antigen Presentation, Interferon Gamma Signaling and PD-1/PD-L1 Cancer Immunotherapy for lung cancer (Supplementary Data 19).

We next examined whether our identified putative cancer susceptibility genes had been reported as predisposition genes (52,72), cancer driver genes (73,74) or CGC (55) (see the ‘Materials and methods’ section). We found eight cancer driver genes and five CGC among previously unreported genes for breast cancer, as well as six cancer driver genes and eight CGC among previously reported genes (Figure 5C). Similarly, for prostate cancer, we found ten cancer driver genes and eight CGC among previously unreported genes, and six cancer driver genes and four CGC among previously reported genes (Figure 5C). For lung cancer, we identified four cancer driver genes and three CGC among previously reported genes. Functional enrichment analysis showed that our identified genes were significantly enriched in those known cancer-related genes with *P* = 0.0044 for breast cancer, *P* = 0.0097 for prostate cancer and *P* = 0.012 for lung cancer (see the ‘Materials and methods’ section).

Gene essentiality in cell proliferation plays a pivotal role in driving key cancer hallmark pathways (75). These essential genes regulate critical processes such as cell growth and survival, and identifying these genes may provide new insights into cancer mechanisms, including the promotion of proliferation, evasion of growth suppressors, or resistance to apoptosis (16,68,69). We next characterized these genes using CRISPR-Cas9 screen silencing data to investigate gene essentiality on cell proliferation in breast (*n* = 45), prostate (*n* = 8) and lung (*n* = 130) cancer relevant cell lines (see the ‘Materials and methods’ section). Using a cutoff of median CERES Score <−0.5 in the above cells, following the previous literature (68,69), we provided strong evidence of essential roles in cell proliferation for 19 previously unreported genes for breast cancer (Figure 5D); 36 unreported genes for prostate cancer (Figure 5E); and two unreported genes for lung cancer (Figure 5F).

### TransTF-TWAS strengthens non-cancer risk gene discovery

To evaluate the generalizability of transTF-TWAS, we conducted additional analysis for brain disorders including SCZ, AD and ASD. By comparison, we also conducted S-PrediXcan and sTF-TWAS for each of the diseases. We found that transTF-TWAS identified more putative susceptibility genes than both sTF-TWAS and S-PrediXcan for AD and ASD. Using ASD as an example, we identified eight putative susceptibility genes from transTF-TWAS at a Bonferroni-corrected *P* < 0.05, while only one and six genes were identified by S-PrediXcan and sTF-TWAS, respectively. The results suggest that our transTF-TWAS approach has broad applicability for enhancing the discovery of disease susceptibility genes (Supplementary Figure S8).

## Discussion

In this study, we demonstrated that the new approach, transTF-TWAS, significantly improved the detection of disease risk genes with increased statistical power and accuracy over other existing TWAS approaches [i.e. sTF-TWAS (25), BGW-TWAS (36), PUMICE (30) and S-PrediXcan (71)]. Under transTF-TWAS framework, we predicted alternative splicing and gene expression and applied these models to large GWAS datasets for breast, prostate and lung cancers. Our analysis revealed a total of 887 putative cancer susceptibility genes, including 465 in regions not yet reported by previous GWAS and 137 in known GWAS loci but not yet reported previously. Many of the newly identified associations have been supported by their oncogenic roles in cancer development (73,74,55), including 88 cancer driver genes, CGC, or those with strong evidence of essential roles in target cancer cell proliferation. These findings provide new insights into the genetic susceptibility of the three common cancers.

Previous TWAS mainly used *cis-*variants in building gene expression models. However, investigation into *trans*-variants has been limited due to the statistical analysis burden of their large numbers. To address this, transTF-TWAS identified many TF-linked *trans-*variants (i.e. an average of 10 for each gene for breast cancer) that significantly contributed to gene expression prediction. Under our analytical framework, we additionally analyzed expression predictions for a total of 4625 genes that can be predicted by both TF-linked translocated and *cis-*located variants using breast normal tissue from the GTEx. We found that *trans*-variants exhibit a greater heritability contribution to gene expression compared to *cis-*variants (Supplementary Figure S9). We also conducted additional tissue-based *trans-*eQTL analysis of *trans-*variants included in our gene expression prediction models. We showed that effect sizes are significantly higher in target tissues (e.g. breast normal tissues) than in non-target tissues, which served as background controls (0.228 versus 0.099 versus 0.056; *P* < 0.05; Supplementary Figure S10). These results provide additional support that the model building for a large majority of genes, by introducing TF-linked *trans-*variants, can facilitate putative causal susceptibility gene discovery via such TF-medicated mechanisms.

For our transTF-TWAS, one critical step was to prioritize TF-linked *trans-*variants for model building based on TF–gene pairs. In our analysis, TF–gene pairs were determined by assigning the binding site to the nearest gene with an additional cutoff of 20 kb. This distance cutoff is based on prior literature, which indicates a decay in TF–gene expression correlation with increasing distance (76). For our defined TF–gene pairs, it is possible that a TF may not play a regulatory role in some of the paired genes due to factors such as experimental conditions or potential errors in peak calling from poor quality ChIP-seq data and analysis. However, the impact of such false TF-target pairs on the gene expression prediction model should be minimal. This is because gene expression is unlikely to be predicted by TF-linked *trans-*variants from false pairs, given the properties of Group Lasso and the subsequent variant selection using the Elastic Net method. Notably, the concept of our approach can be extended to identify TF-linked *trans-*variants through integration of additional single cell omics data, such as long-distance epigenetic signals (>1 Mb) like distal chromatin–chromatin interactions, enhancer-gene links, enhancer-gene correlations and *trans-*eQTLs.

Deep learning approaches, such as Enformer—a highly sophisticated deep neural network model—have been utilized to enhance variant effect predictions on gene expression by leveraging extensive epigenetic datasets, including TF binding, chromatin marks and transcription profiles from various human tissues and cells (77,78). Despite its success in annotating regulatory variants, its performance has been limited in explaining variations in gene expression across individuals (79). Our approach focuses on whether TF regulates the expression of the downstream gene, determining whether its associated variants are included in the model. This design is specifically tailored to address the challenge of having too many terms when analyzing *trans-*variants. Further integration of our TF-linked *trans-*variants with this deep learning framework into TWAS could significantly enhance the identification of risk genes in future studies.

In conclusion, we demonstrated that our transTF-TWAS, by integrating TF-linked *trans-*variants with TWAS, significantly improved disease susceptibility gene discovery and advanced our understanding of complex human diseases, including cancers. Our study also highlighted several genetically driven key regulators and their associated regulatory networks underlying disease susceptibility.

## Supplementary Material

gkae1035_Supplemental_Files

## Data Availability

Supplementary Data 1 provides the download information for the summary statistics of GWAS data for breast cancer, prostate cancer, lung cancer and three brain disorders (SCZ, ASD and AD); the epigenetic data, including ChIP-seq data of transcription factors, DHSs, enhancer, promoter, 3D genomics informed regions, enhancer gene links and eQTLs used in this study; and the functional annotation data, including target cancer related genes, CGC and cancer driven genes. Gene expression and alternative splicing data generated in breast, prostate, lung and brain tissue were downloaded from GTEx consortium, and the individual-level genotype was downloaded from dbGaP (https://www.ncbi.nlm.nih.gov/projects/gap/cgi-bin/study.cgi?study_id=phs000424.v8.p2). Access to gene expression data in normal breast tissue from Kome is available via the dbGaP (associated with NIH-funding R0CA235553). Gencode annotation (v26.GRCh38) was downloaded from https://www.gencodegenes.org/human/release_26.html. The data from the 1000 Genomes Project was downloaded through the website, https://www.genome.gov/27528684/1000-genomes-project. For data of essentiality for proliferation and survival of cancer cells, we downloaded two comprehensive datasets, including ‘sample_info.csv’ and ‘Achilles_gene_effect.csv’ from the DepMap portal (Supplementary Data 1). Remaining data sources and results are provided within the Article or Supplementary Data. The developed pipeline and main source R codes used in this work are available from the Github website: https://github.com/theLongLab/transTF-TWAS or https://github.com/XingyiGuo/transTF-TWAS/ or https://github.com/XingyiGuo/transTF-TWAS/ and we have deposited the codes to Zenodo at 10.5281/zenodo.10780753.
