# Correction to ‘Enhancing disease risk gene discovery by integrating transcription factor-linked *trans*-variants into transcriptome-wide association analyses’

**DOI:** 10.1093/nar/gkae1239

**Published:** 2024-12-10

This is a correction to: Jingni He, Deshan Perera, Wanqing Wen, Jie Ping, Qing Li, Linshuoshuo Lyu, Zhishan Chen, Xiang Shu, Jirong Long, Qiuyin Cai, Xiao-Ou Shu, Zhijun Yin, Wei Zheng, Quan Long, Xingyi Guo, Enhancing disease risk gene discovery by integrating transcription factor-linked *trans*-variants into transcriptome-wide association analyses, *Nucleic Acids Research*, 2024; https://doi.org/10.1093/nar/gkae1035

The authors would like to replace Figure 1 as this was a placeholder version during proofreading.

Figure 1 has now been replaced with the updated version:


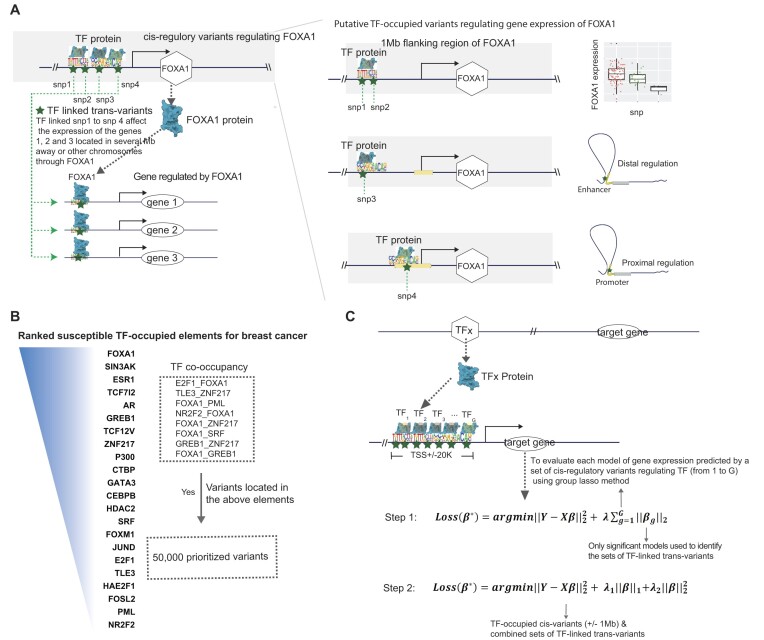


instead of:


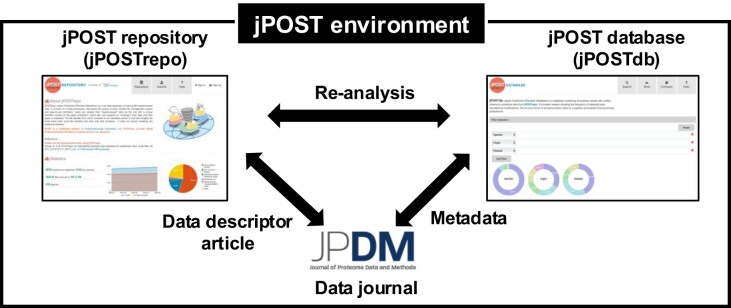


This change does not affect the results, discussion and conclusions presented in the article.

The published article has been updated.

