# Supplementary material for: Enhancing disease risk gene discovery by integrating transcription factor-linked trans-variants into transcriptome-wide association analyses
Source: Nucleic Acids Res. 2024 Nov 13;53(1):gkae1035. doi: 10.1093/nar/gkae1035 (PMC11724290; doi:10.1093/nar/gkae1035)
Supplement: gkae1035_Supplemental_Files [file gkae1035_supplemental_files.zip › Supplementary_Materials_08272024.pdf]

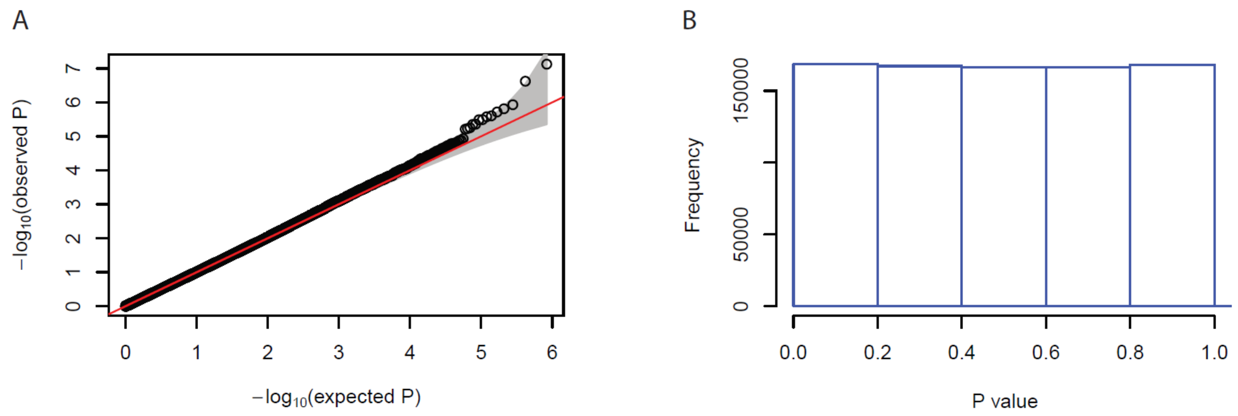

Supplementary Figure S1. P-values plotted against the null expectation in a quantile-quantile plot under null simulations. A. QQ plot showing P-values from transTF-TWAS analyses using 100 sets of prioritized cis-located and trans-located genetic variants. B. Histogram of P-values showing a uniform distribution as expected under the null. The P-values are the raw P-values from the Z score test from transTF-TWAS.

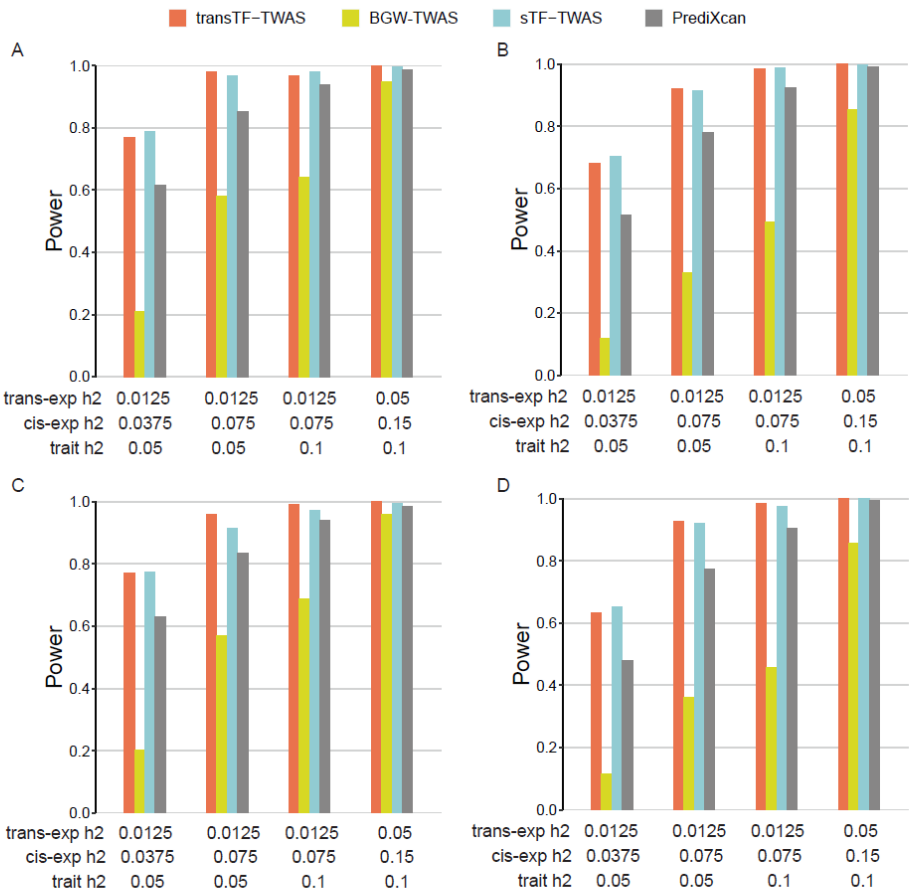

Supplementary Figure S2. Power comparison under pleiotropy and causality scenarios for gene expression heritability that are weakly contributed by TF-linked trans-located variants. Power is indicated on the y-axis. All panels are results under an additive genetic architecture, with differing trans-variants expression heritability, cis- variants expression heritability and trait heritability denoted below each panel. A-B. under pleiotropy scenario. A. 20 causal genetic variants. B. 50 causal genetic variants. C-D. under causality scenario. C. 20 causal genetic variants. D. 50 causal genetic variants.

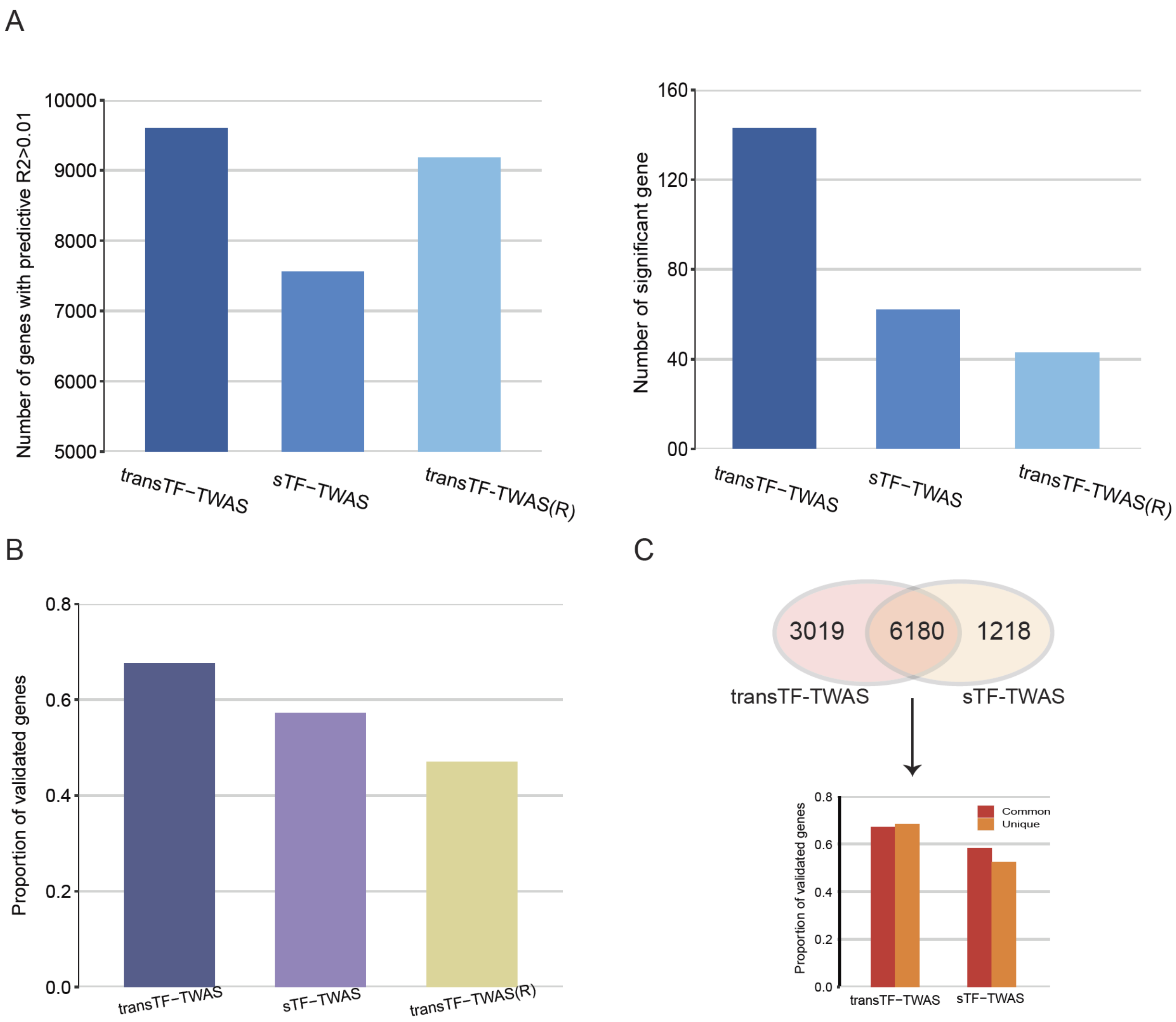

Supplementary Figure S3. Validation using breast normal tissues from KOME. A. The left bar chart showed the number of genes with predictive $R^2 > 0.01$ among transTF-TWAS, sTF-TWAS and transTF-TWAS (R). The right bar chart showed the number of significant gene among transTF-TWAS, sTF-TWAS and transTF-TWAS (R). The number of significantly identified genes was indicated at a Bonferroni-corrected $P < 0.05$. B-C. The proportion of validated genes means the number of genes with predictive $R^2>0.01$ in both GTEx and Komen data divided the total number of genes. B. The group bar chart showed the proportion of validated genes among transTF-TWAS, sTF-TWAS and transTF-TWAS(R). C. The Venn diagram showed the number of uniquely or commonly identified genes with predictive $R^2>0.01$ between transTF-TWAS and sTF-TWAS using Komen data. The group bar chart showed the comparison of the proportion of validated genes between the uniquely and commonly identified genes for transTF-TWAS and sTF-TWAS.

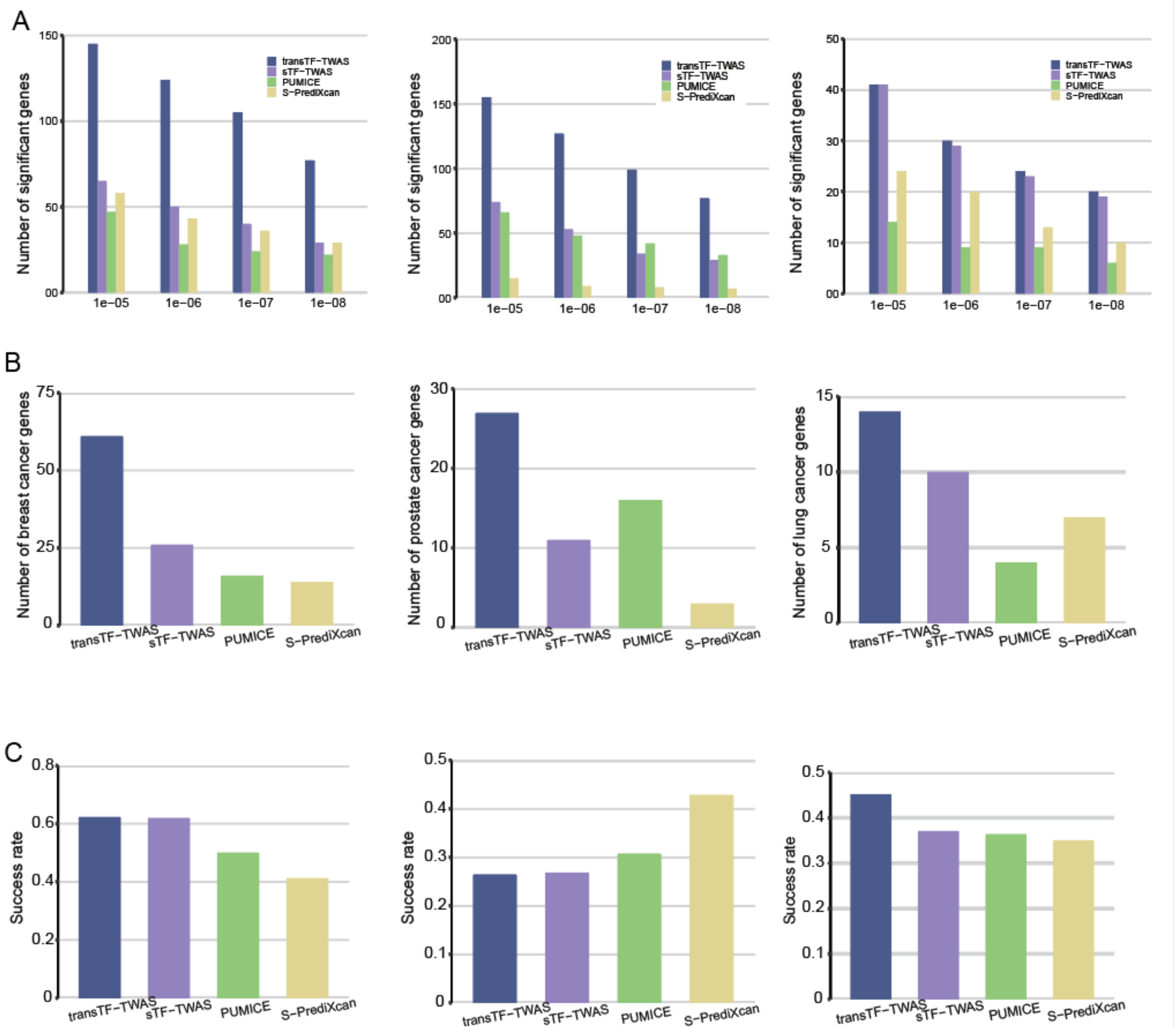

Supplementary Figure S4. Comparison of gene-trait associations between transTF-TWAS with other TWAS approaches (sTF-TWAS, PUMICE, and S-PrediXcan) for breast, prostate and lung cancer. A. Bar chart showing the number of genes identified from transTF-TWAS and other TWAS approaches under various *P*-value cutoffs (i.e., $P <$ 1e-05, 1e-06, 1e-07, and 1e-08). The P-values are the nominal P-values from the Z score test from TWAS. B. Bar chart showing a comparison between the total number of target cancer related genes among transTF-TWAS and other TWAS approaches. C. Bar chart showing a comparison of the proportion (success rate) of target cancer related gene among transTF-TWAS and other TWAS approaches, relative to the total number of genes identified from the set.

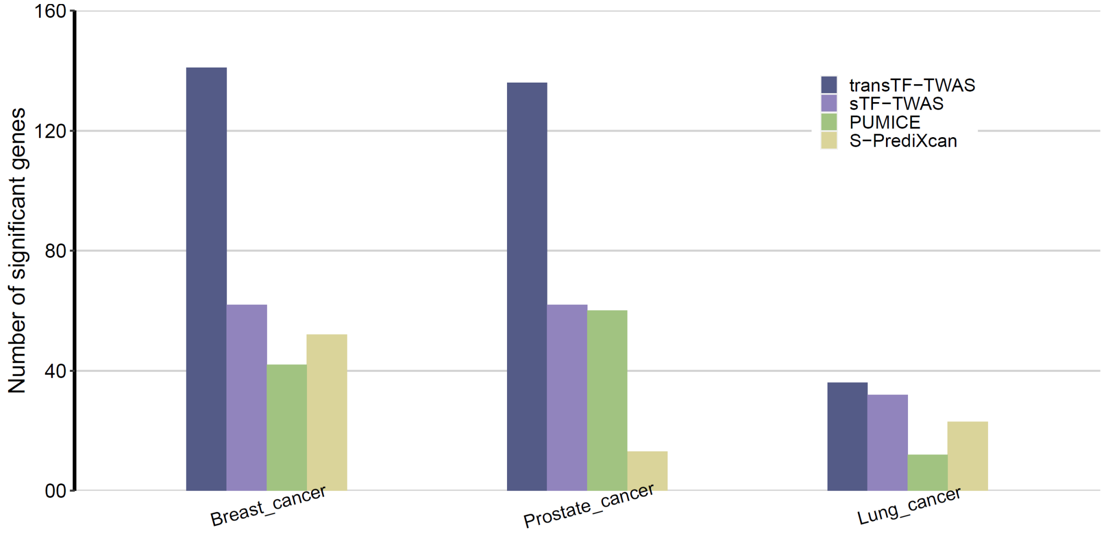

Supplementary Figure S5. Comparisons of transTF-TWAS and other TWAS approaches in breast, prostate and lung cancers. The group bar chart showed the number of significant genes among transTF-TWAS, sTF-TWAS, PUMICE and S-PrediXcan for breast, prostate and lung cancers. The number of significantly identified genes was indicated at a Bonferroni-corrected P<0.05.

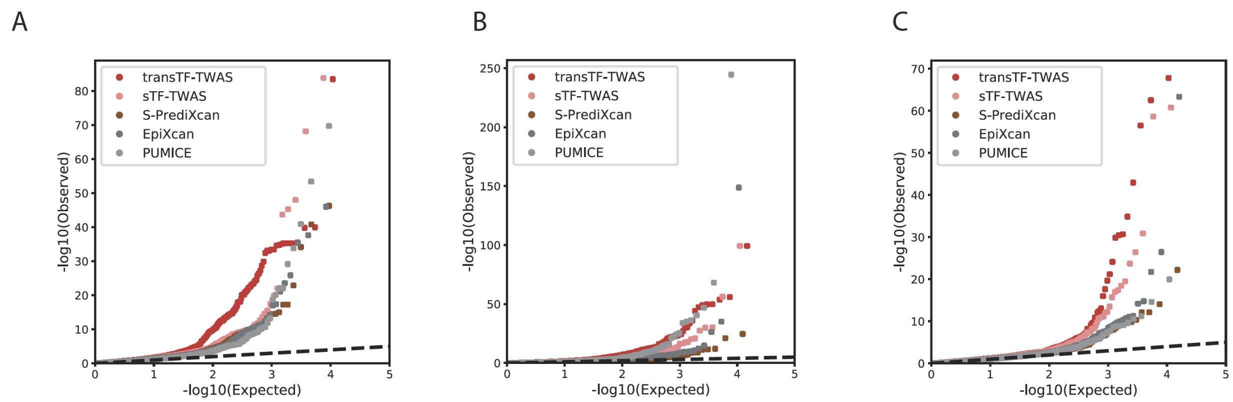

Supplementary Figure S6. Association *P* values plotted against the null expectation in a quantile-quantile plot. Each plot showed the comparison of gene-trait associations among transTF-TWAS and other TWAS approaches, including sTF-TWAS, S-PrediXcan, EpiXcan and PUMICE. The dash line shows the null expected distribution of P values. The *P*-values are the raw *P*-values from the Z score test from TWAS. A. breast cancer. B. prostate cancer. C. lung cancer.

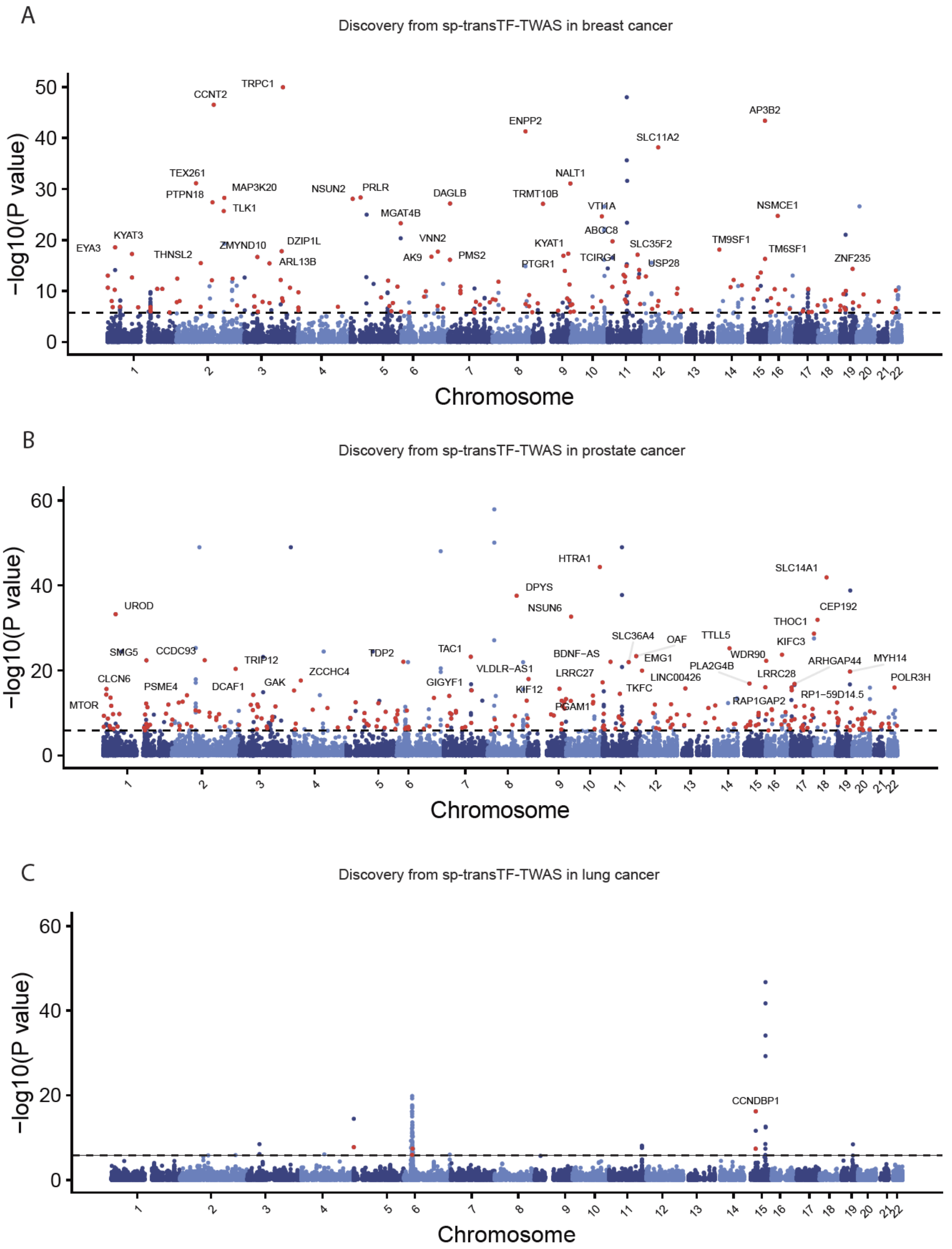

Supplementary Figure S7. Putative susceptibility genes identified by sp-transTF-TWAS. Manhattan plots showing associations identified from sp-transTF-TWAS. Red dots indicated all newly identified susceptibility genes, and the grey dashed line refers to Bonferroni-corrected P < 0.05. The newly identified putative susceptibility genes with $P < 10^{-15}$ were highlighted. The P-values are the raw P-values from the Z score test from TWAS (two-sided). A. breast cancer. B. prostate cancer. C. lung cancer.

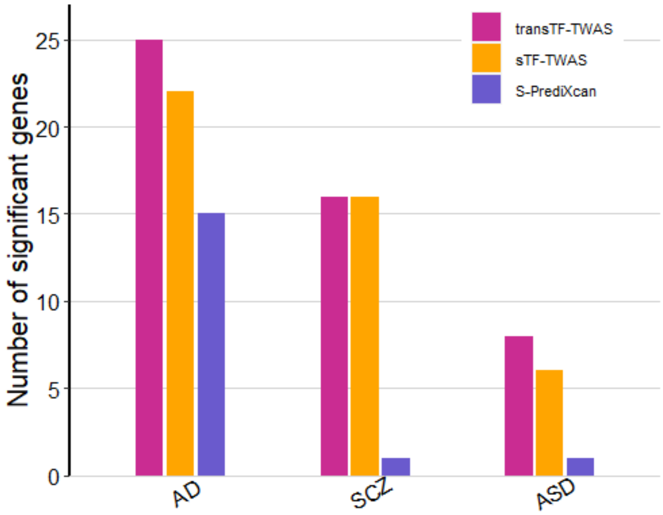

Supplementary Figure S8. Comparisons of transTF-TWAS, sTF-TWAS and S-PrediXcan in brain disorders. The number of significantly identified genes was indicated at a Bonferroni-corrected $P<0.05$. SCZ: schizophrenia; AD: Alzheimer's disease; ASD: autism spectrum disorder.

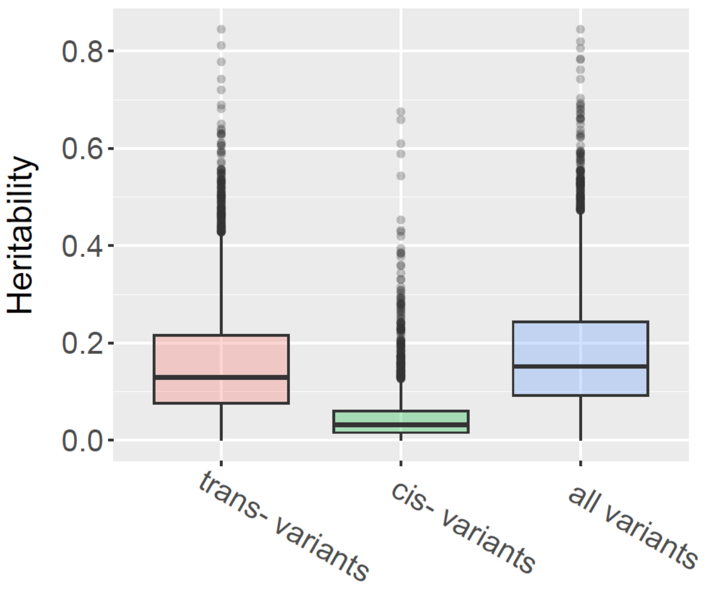

Supplementary Figure S9. Gene expression heritability contributed by TF-linked trans-located variants, cis-variants, and both translocated and cis-variants. On the X-axis, "trans-variants" and "cis-variants" refer to gene expression heritability contributed by trans-located and cis- variants, respectively; "all variants" encompasses heritability contributed by both trans-located and cis-variants. The heritability on the y axis was calculated based on the GCTA package (PMID: 21167468).

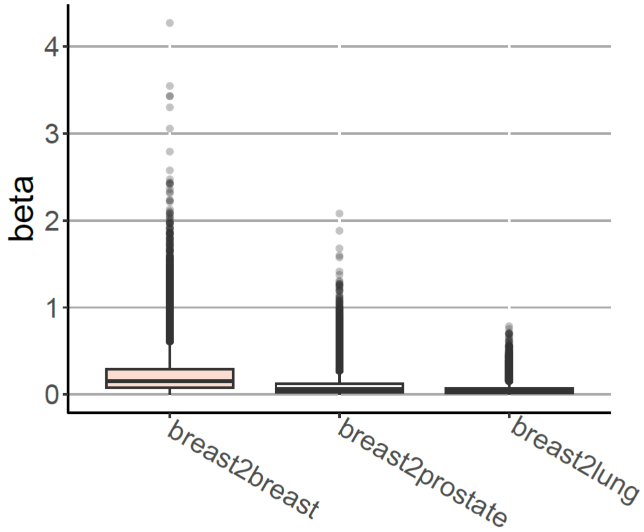

Supplementary Figure S10. Comparison of Trans-eQTL analysis in breast normal tissues and non-target tissues (i.e., non-breast normal tissues). The trans-eQTL analyses were conducted for trans-variants, which were prioritized by transTF-TWAS model of a given gene for breast tissue. Based on the same set of these variants, we defined 'breast2breast', ‘breast2prosate’ and ‘breast2lung’ based on the trans-eQTL analysis using data from breast, prostate and lung tissues. The boxplot illustrates significantly higher absolute beta values in target tissues compared to non-target tissues.
